# First-Line* Helicobacter pylori* Eradication in Patients with Chronic Kidney Diseases in Taiwan

**DOI:** 10.1155/2017/3762194

**Published:** 2017-12-11

**Authors:** Chih-Ming Liang, Chien-Hua Chiu, Hsing-Ming Wang, Wei-Chen Tai, Chih-Chien Yao, Cheng-En Tsai, Chung-Mou Kuo, Yi-Chun Chiu, Keng-Liang Wu, Chen-Hsiang Lee, Kai-Lung Tsai, Chih-Fang Huang, Seng-Kee Chuah

**Affiliations:** ^1^Division of Hepatogastroenterology, Kaohsiung Chang Gung Memorial Hospital and Chang Gung University College of Medicine, Kaohsiung, Taiwan; ^2^Division of Nephrology, Kaohsiung Chang Gung Memorial Hospital and Chang Gung University College of Medicine, Kaohsiung, Taiwan; ^3^Division of Infectious Diseases and General Medicine, Department of Internal Medicine, Kaohsiung Chang Gung Memorial Hospital and Chang Gung University College of Medicine, Kaohsiung, Taiwan; ^4^Division of Colon and Rectal Surgery, Department of Surgery, Kaohsiung Chang Gung Memorial Hospital and Chang Gung University College of Medicine, Kaohsiung, Taiwan; ^5^Department of Family Physician, Kaohsiung Chang Gung Memorial Hospital and Chang Gung University College of Medicine, Kaohsiung, Taiwan

## Abstract

**Aims:**

Patients with chronic kidney disease (CKD) and* Helicobacter pylori* (*H. pylori*) infection have a higher incidence of gastroduodenal diseases and therefore are recommended to receive eradication therapies. This study aimed to assess the efficacy of a 7-day standard triple therapy in patients with CKD (eGFR < 60 ml/min/1.73 m^2^) and to investigate the clinical factors influencing the success of eradication.

**Methods:**

A total of 758 patients with* H. pylori* infection receiving a 7-day standard first-line triple therapy between January 1, 2013, and December 31, 2014, were recruited. Patients were divided into two groups: CKD group (*N* = 130) and non-CKD group (*N* = 628).

**Results:**

The eradication rates attained by the CKD and non-CKD groups were 85.4% and 85.7%, respectively, in the per-protocol analysis (*p* = 0.933). The eradication rate in CKD stage 3 was 84.5% (82/97), in stage 4 was 88.2% (15/17), and in those who received hemodialysis was 87.5% (14/16). There were no significant differences in the various stages of CKD (*p* = 0.982). The adverse events were similar between the two groups (3.1% versus 4.6%, *p* = 0.433). Compliance between the two groups was good (100.0% versus 99.8%, *p* = 0.649). There was no significant clinical factor influencing the* H. pylori* eradication rate in the non-CKD and CKD groups.

**Conclusions:**

This study suggests that the* H. pylori* eradication rate and adverse rate in patients with CKD are comparable to those of non-CKD patients.

## 1. Introduction

A high incidence of chronic kidney disease (CKD) in Taiwan has been reported in the United States Renal Data System 2010 Annual Data Report [1]. This is a threat to the national health of the people. As per Hwang et al.'s report [2], the overall awareness of CKD is low in Taiwan: 9.7% for CKD stages 1–3 and 3.5% for stages 1–5. People need to be educated to address the risk factors associated with CKD, such as diabetes mellitus, glomerulonephritis, hypertension, older age, smoking, obesity, herbal medicine use, chronic lead exposure, and hepatitis C in public health program. Patients with CKD often have a higher incidence of peptic ulcer disease (PUD) than the general population, with a substantially increased PUD risk during the 10 years following diagnosis [3, 4]. Furthermore, CKD patients have higher peptic ulcer bleeding (PUB) complications, such as recurrent bleeding, infection, and mortality than the general population [5–8].

*Helicobacter pylori* (*H. pylori*) plays an important role in the development of chronic gastritis, gastric ulcers, duodenal ulcers, and gastric cancer [9–12]. According to the Taiwan National Health Insurance Research Database, although there is a lower* H. pylori* infection rate in patients with CKD (58.5%) and ESRD (56.2%) and PUD than in those with PUD without CKD (70.2%) [13], early* H*.* pylori* eradication (≤90 days) is highly suggested because it is associated with a protective role against the exacerbation of kidney malfunction and overall mortality [14].

The metabolism of certain drugs such as antibiotics could be altered in patients with CKD. Therefore, the influence on* H. pylori* eradication rate and adverse events of triple therapy need to be further studied. To our knowledge, the reports on* H. pylori* eradication in patients with CKD are scarce in Taiwan. This study aimed to assess the efficacy of a 7-day standard triple therapy in patients with CKD undergoing hemodialysis and to investigate the clinical factors influencing the success of eradication.

## 2. Materials and Methods

### 2.1. Patients

**Figure 1:**
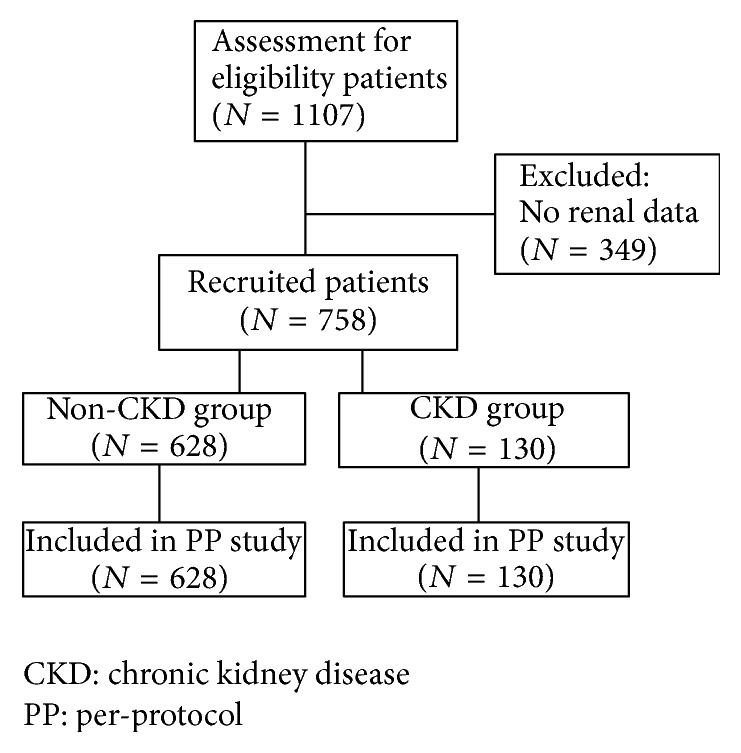
Disposition of patients.

A total of 1107 patients infected with* H. pylori* receiving a first-line triple therapy were retrospectively studied between January 1, 2013, and December 31, 2014, at outpatient clinics in Kaohsiung Chang Gung Memorial Hospital, Taiwan. Of these patients, 758 were recruited in the per-protocol (PP) study after excluding 349 patients due to incomplete chart recording. All patients were at least 18 years of age and had received endoscope examinations that showed either peptic ulcers or gastritis. Patients were then divided into two groups: CKD group (*n* = 130) and non-CKD group (*n* = 628) (Figure 1). Patients in the non-CKD group received a standard triple therapy [proton-pump inhibitor (PPI) twice daily, 500 mg clarithromycin twice daily, and 1 g amoxicillin twice daily for 7 days], whereas ESRD patients in the CKD group received PPI twice daily and half the dose of clarithromycin and amoxicillin twice daily for 7 days.

Based on the revised 4-variable MDRD Study equation [15], all individuals with a glomerular filtration rate (GFR) of <60 ml/min/1.73 m^2^ for 3 months were classified as having CKD, irrespective of the presence or absence of kidney damage. The rationale for including these individuals was that reduction in kidney function to this or lower level represents loss of half or more of the adult level of normal kidney function, which may be associated with a number of complications such as the development of cardiovascular diseases [16].

*H. pylori* eradication failure was confirmed if patients had either one positive ^13^C-UBT or any two positive results of the rapid urease test, histology, and culture after first-line eradication therapy. According to our hospital requirements, all registered patients were followed up to assess drug compliance and adverse effects as soon as they finished their medications. These patients then underwent either an endoscopy or a urea breath test 4–8 weeks later. Poor compliance was defined as failure to finish 80% of all medication due to adverse effects [17].

Demographic information including age, sex, social history of smoking, alcohol consumption, previous peptic ulcer history, and laboratory data (AST, ALT, total bilirubin, albumin, BUN, Cr, sodium, potassium, calcium, hemoglobin, cholesterol, and triglyceride) were collected via electrical medical records. This study was approved by the Institutional Review Board and Ethics Committee of Chang Gung Memorial Hospital, Taiwan (IRB 201700772B0). The Ethics Committee waived the requirement for informed consent, and each patient's medical records were anonymized and not identified before access. All patients provided their written informed consent before endoscopic interventions.

### 2.2. Statistical Analysis

The primary outcome variables were eradication rate, presence of adverse events, and level of patient compliance. Using the Statistical Package for the Social Sciences version 18 (SPSS, Chicago, IL, USA), Chi-square tests with or without Yates' correction for continuity and Fisher's exact tests were used when appropriate to compare the major outcomes between groups. Eradication rates were analyzed by PP approaches. The PP analysis excluded patients with unknown* H. pylori* status following therapy and those with major protocol violations. A *p* value < 0.05 was considered statistically significant. To determine the independent factors that affected treatment response, the clinical and laboratory parameters were analyzed by univariate and multivariate analyses.

## 3. Results

Figure 1 shows patient flowchart. The demographic data of the two groups are summarized in Table 1. In comparison to patients in the non-CKD group, patients in the CKD group were older (68.6 ± 10.0 versus 58.2 ± 11.3, *p* < 0.001) and had a higher incidence of peptic ulcer history (30% versus 16.9%, *p* = 0.001), higher BUN levels (33.2 ± 22.2 mg/dl versus 14.2 ± 20.0 mg/dl, *p* < 0.001), higher potassium levels (5.9 ± 2.7 mEq/L versus 4.2 ± 2.9 mEq/L, *p* = 0.024), lower chloride levels (97.5 ± 24.4 mEq/L versus 104.5 ± 10.4 mEq/L, *p* = 0.029), lower hemoglobin levels (11.4 ± 2.2 g/dL versus 13.7 ± 3.1 g/dL, *p* < 0.001), and lower cholesterol levels (168.1 ± 29.5 mg/dL versus 189.2 ± 38.8 mg/dL, *p* = 0.001). The eradication rates attained by the CKD and non-CKD groups were 85.4% (111/130) and 85.7% (538/628), respectively, in the PP analysis (*p* = 0.933) (Table 2). The eradication rates in the different stages of CKD were as follows: 84.5% in stage 3, 88.2% in stage 4, and 87.5% in hemodialysis (*p* = 0.982) (Table 3).

### 3.1. Adverse Events and Compliance

Since amoxicillin and clarithromycin are primarily eliminated via the renal route, these antibiotics need a dosage adjustment based on GFR in patients with renal failure. Therefore, we prescribed the half-dose triple therapy with clarithromycin and amoxicillin to eradicate* H. pylori* in patients with ESRD. The adverse events were similar between the two groups (3.1% versus 4.6%, *p* = 0.433) (Table 2). These adverse events included abdominal pain, constipation, diarrhea, dizziness, headache, and nausea/vomiting. However, these adverse events were mild and did not disturb the patients' daily activities. Both groups had good drug compliances (100% in the CKD group versus 99.8% in the non-CKD group, *p* = 0.649). Only one patient did not complete the triple eradication therapy in the non-CKD group: a 68-year-old male patient who stopped taking medications after developing severe vomiting following the triple eradication therapy at day 3.

### 3.2. Factors Influencing the Efficacy of Anti-*H. pylori* Therapy

In the univariate analysis of the CKD and non-CKD groups, there was no significant clinical factor influencing the* H. pylori* eradication rate in patients with CKD (Table 4).

## 4. Discussion

Patients with chronic renal failure generally have a higher incidence of gastrointestinal symptoms than the general population, which is associated with not only* H. pylori* infection but also high urea levels, impairment of gastrointestinal motility, amyloid protein deposition [18, 19], and decreased sensory disturbance. Furthermore, patients with CKD are at a higher risk of gastric mucosal damage than those with normal renal function because of coexisting comorbidities such as diabetes and coronary artery diseases [20, 21], hypergastrinemia [22], and poor systemic circulation, resulting in enhanced inflammation of the gastrointestinal mucosa.

Till now, the eradication of* H. pylori* infection was recommended as a critical step in preventing and treating PUD not only in patients with normal renal function but also in those with renal failure [23–25]. Although triple regimen showed disappointing results (80%) in Taiwan due to high clarithromycin resistance (22%) and is not recommended by Taiwan consensus, it still remains the most widely used 1st-line* H. pylori* eradication therapy [26, 27]. Since medical expenses in Taiwan are generally covered by the Taiwanese National Health Insurance administration, standard triple therapy is still the recommended first-line empiric regimen. Therefore, we recommend replacing this standard triple therapy with a 4-drug combination treatment. This course may be sequential, concomitant, or hybrid and may involve extension of the triple therapy to 14 days to improve the eradication rates [28–31].

Several studies reported various eradication rates by triple therapy regimens ranging from 72.7% to 94.1% in hemodialysis-dependent patients [32–35]. In the clinical trial by Makhlough et al., the eradication rate of a standard triple therapy in CKD stage 3 was 50% (1/2), in CKD stage 4 was 75% (3/4), and in hemodialysis was 80% (12/15) [36]. In our study, which included larger case numbers than previous studies on CKD, the eradication rate in CKD stage 3 was 84.5% (82/97), in CKD stage 4 was 88.2% (15/17), and in hemodialysis was 87.5% (14/16). There is no significant difference in the various stages of CKD (*p* = 0.982). Therefore, the successful rate of eradication was similar in the different stages of CKD.

In this study, the adverse events were similar between the two groups (3.1% versus 4.6%, *p* = 0.433). The most common side effect was abdominal bloating. The physician should not be afraid of the adverse effect of these anti-*H. pylori* drugs. Instead, they should be more motivated to eradicate* H. pylori* in patients with CKD considering the potential complications and mortality in these patients who suffer from PUB [13, 14]. With respect to the dosage, Ehsani Ardakani et al. reported that half-dose triple therapy with clarithromycin, amoxicillin, and omeprazole is as effective as full-dose triple therapy in patients with ESRD [37]. Also, they found that more patients developed a bitter taste in their mouths as well as abdominal distension in the full-dose group (73.6% versus 39.7%, *p* = 0.014) compared with the half-dose group (41.2% and 18.3%, *p* = 0.04). To lower toxicity, adverse events, and cost of the half-dose regimen in this subset of patients, adjusting the dose of the eradication protocol according to the renal function of patient is advised [38, 39].

There are some limitations to this study. First, it is a single-center retrospective study. Second, the number of patients with CKD is small. Third, no information on antibiotic resistance to* H. pylori* was available.* H. pylori* culture was not routinely conducted before triple therapy. Fourth, the follow-up of* H. pylori* eradication status was conducted 4–8 weeks after therapy. Checking an effective response at 4 weeks seemed too early since this may lead to pseudonegative results especially at 4 weeks after the end of therapy.

## 5. Conclusion

This study suggests that* H. pylori* eradication rate and adverse events in the CKD group were comparable to those of the non-CKD group. Neither group achieved >90% eradication rates with the standard triple therapy. Therefore, further studies are warranted to search for an optimal regimen for treating patients with CKD and* H. pylori* infection.

## Figures and Tables

**Table 1:** Demographic data and endoscopic appearances of the two patient groups.

	CKD (*n* = 130) *n* (%)	Control non-CKD (*n* = 628) *n* (%)	*p* value
Age (year) (mean ± SD)	68.6 ± 10.0	58.2 ± 11.3	<0.001
Sex (male/female)	53/77	303/325	0.120
Smoking, *n* (%)	14 (10.8)	94 (15.0)	0.213
Alcohol consumption, *n* (%)	14 (10.8)	101 (16.1)	0.124
Previous history of peptic ulcer, *n* (%)	39 (30.0)	106 (16.9)	0.001
Endoscopic findings, *n* (%)			
Gastritis	35 (26.9)	214 (34.1)	
Gastric ulcer	60 (46.2)	190 (30.3)	0.002
Duodenal ulcer	22 (16.9)	169 (26.9)	
Gastric and duodenal ulcer	13 (10.0)	55 (8.8)	
*Laboratory data (mean ± SD)*			
AST (U/L)	29.1 ± 13.8	28.8 ± 21.4	0.921
ALT (U/L)	25.2 ± 15.4	31.4 ± 29.4	0.051
Total bilirubin (mg/dl)	0.7 ± 0.5	1.9 ± 1.3	0.564
Albumin (g/dl)	4.2 ± 0.3	4.4 ± 0.6	0.089
Total protein (g/dl)	7.0 ± 0.4	7.7 ± 6.8	0.716
BUN (mg/dl)	33.2 ± 22.2	14.2 ± 20.0	<0.001
Na (mEq/L)	137.0 ± 18.9	139.3 ± 11.2	0.231
K (mEq/L)	5.9 ± 2.7	4.2 ± 2.9	0.024
Ca (mEq/L)	9.3 ± 0.8	11.0 ± 13.0	0.480
Cl (mEq/L)	97.5 ± 24.4	104.5 ± 10.4	0.029
GFR (ml/min/1.73 m^2^)	39.0 ± 16.6	90.1 ± 19.3	<0.001
Hemoglobin (g/dL)	11.4 ± 2.2	13.7 ± 3.1	<0.001
Cholesterol (mg/dl)	168.1 ± 29.5	189.2 ± 38.8	0.001
Triglyceride (mg/dl)	152.6 ± 80.0	126.4 ± 79.9	0.055

CKD: chronic kidney disease, AST: aspartate aminotransferase; ALT: alanine aminotransferase, BUN: blood urea nitrogen, GFR: glomerular filtration rate, Na: sodium, K: potassium, Cl: chloride, and Ca: calcium.

**Table 2:** Major outcomes of eradication therapy.

	Eradication rate		
	CKD (*n* = 130)	Non-CKD (*n* = 628)	*p* value
Per-protocol	85.4% (111/130)	85.7% (538/628)	0.933
Adverse event	3.1% (4/130)	4.6% (29/628)	0.433
Compliance	100.0% (130/130)	99.8% (627/628)	0.649

CKD: chronic kidney disease.

**Table 3:** *Helicobacter pylori* eradication rates in different stages of kidney disease.

CKD stage	Stage 3 (*n* = 97)	Stage 4 (*n* = 17)	Hemodialysis (*n* = 16)	Total (*n* = 130)	*p* value
Eradication rate	84.5% (82)	88.2% (15)	87.5% (14)	85.4% (111)	0.982

CKD: chronic kidney disease.

**Table 4:** Univariate analysis of the clinical factors influencing the efficacy of *H. pylori* eradication.

Principle parameter		CKD (*n* = 130)		*p* value	Non-CKD (*n* = 628)		*p* value
		Case number	Eradication rate (%)		Case number	Eradication rate (%)	
Age	≥60 years	90/107	84.1	0.376	216/309	69.9	0.397
	<60 years	21/23	91.3		277/319	86.8	
Sex	Female	66/77	85.7	0.898	286/325	88.0	0.084
	Male	45/53	84.9		252/303	83.2	
Smoking	(−)	98/116	84.5	0.402	460/534	86.1	0.420
	(+)	13/14	92.9		78/94	83.0	
Alcohol consumption	(−)	98/116	84.5	0.402	455/527	86.3	0.274
	(−)	13/14	92.9		83/101	82.2	
Previous history of peptic ulcer	(−)	98/116	84.5	0.871	448/522	85.8	0.806
	(+)	13/14	92.9		90/106	84.9	

Laboratory data							
Na (mEq/L)	≥130	44/53	83.0	0.524	285/327	87.2	0.348
	<130	2/2	100		6/6	100	
K (mEq/L)	≥5	10/11	90.9	0.616	6/6	100	0.337
	<5	46/54	85.2		312/360	86.7	
Ca (mEq/L)	≥8	25/29	86.2	0.574	176/195	90.3	0.743
	<8	2/2	100		1/1	100	
Albumin (g/dl)	≥3.5	29/34	85.3	0.679	194/218	89.0	0.321
	<3.5	1/1	100		8/8	100	
BUN (mg/dl)	≥20	37/46	76.1	0.102	29/32	90.6	0.552
	<20	21/22	95.4		253/291	86.9	
GFR (ml/min/1.73 m^2^)	≥30	82/97	84.5	0.639	538/628	85.7	—
	<30	29/33	87.9		0		
Cholesterol (mg/dl)	≥200	6/6	100	0.237	97/110	88.2	0.776
	<200	29/36	80.6		175/201	87.1	
Triglyceride (mg/dl)	≥150	14/17	82.4	0.687	76/85	89.4	0.419
	<150	20/23	87.0		183/213	85.9	
Hemoglobin (g/dL)	≥10	67/79	84.8	0.922	408/473	86.3	0.745
	<10	21/25	84.0		30/34	88.2	

CKD: chronic kidney disease, BUN: blood urea nitrogen, GFR: glomerular filtration rate, Na: sodium, K: potassium, Cl: chloride, and Ca: calcium.
